# Mechanical Dentistry

**Published:** 1886-08

**Authors:** P. T. Dedman

**Affiliations:** Lebanon


### Mechanical Dentistry.

(Read before the Kentucky State Dental Society.)

BY. P. T. DEDMAN, LEBANON.

In appearing before you to-day it is not my expectation to throw any light upon the subject of Mechanical Dentistry, but I desire to perform a duty that this Association has assigned me, and to bring about, if possible, a discussion upon this all important subject. The profession of late years has ignored this branch almost entirely, they meet annually, the subject is mentioned and that is about all. I regret my inability to present to you any new theories or ideas by which to promote a science so important to our profession, but one that has gradually fallen into disrepute ever since the introduction of cheap bases. Of the origin of Mechanical Dentistry, we know but little, we have evidence of its existance in remote ages, but when and by whom it was first introduced we do not know. In the olden time little attention was paid to dentistry in consequence of the expense attending it, and the scarcity of enlightened dentists, but as the demand increased we find dentists rising up all over the land, whose patient investigations, and whose untiring experiments have been crowned with the grandest success until the science has at length been raised to its present dignity and importance. It was not until the introduction of cheap bases that artificial teeth were so generally used. When metallic plates only were used, it required the master's skill and a knowledge of metals to construct a plate, but now it is an easy matter for the beginner to learn in a few months to put up a set of teeth on celluloid or rubber. While we can but admit that the introduction of cheap bases has been a blessing to a great number of our patients; placing artificial teeth within the reach of the poorer classes, yet we do believe that it has been a misfortune to the profession and to some of our patients, it has indeed, been a great calamity that they were ever introduced. There are some members of our profession, gentlemen, whose lack of manual skill prevents them from recommending a base they are not able to construct. We, as dentists, being educators of our patients, are greatly to blame for the extensive use of cheap materials. They come to us for advice, we tell them what is best and they accept it. Some will say the mass of the people demand cheap material, and why not gratify them. I do not object to their having them, but I do object to telling them it is superior to anything else. The demand is sufficient without misrepresentation.

I am not here to condemn the use of cheap bases by any means, they serve a good purpose and should be used when circumstances demand it. I only condemn useing them to the exclusion of what is a great deal better for those who can afford to pay for for them. I believe, however, that there have been many teeth extracted that would not have been had we known nothing of the cheap bases; the cheapness of the material has been an inducement for many to have teeth extracted (that could have been easily saved), for the purpose of inserting artificial ones, and the unscrupulous charlatan, with but a few dollars as an incentive, has induced patients to have teeth extracted when there was no necessity for it whatever.

The uneducated are thus robbed of organs that can only be replaced by a substitute which is but poor at best, and then one of the best substitutes discarded because forsooth the dentist does not care to go to a little trouble, or is ignorant of what should be done. Since the time when teeth were carved out of bone down to the days of continuous gum work, perfection in Mechanical Dentistry has never been attained. The platinum plate with continuous gum is the most artistic and possibly one of the best plates now in use, but the expense attending its construction places it beyond the reach of a majority; and besides, there are but few dentists, even if they are capable, ever do this kind of work. I know but little of "bridge work," but believe if it be possible to keep it thoroughly clean, that in cases where it could be used successfully that nothing would be equal to it.

It is my candid opinion that the evil effects arising from the use of vegetable bases are more serious than we imagine, and I think, after our patients become better educated in regard to dentistry this will deter many from using them, and metallic plates will become more generally used by those who can afford them.

In an article lately written on Bridge Work, the author, in speaking of the inflammation and bad results generally brought about by the wearing of non-conducting materials covering the roof of the mouth, says, in order to digest certain kinds of food, in fact all of the carbo-hydrates, such as starch, sugar, oil, gela tine, gum, and flesh, the combined secretions of the follicles and salivary glands are needed, and these could not be had where the roof of the mouth was covered; consequently, if we are not able to digest these as nature designed, the injury liable to occur, physically and mentally is incalculable. Let us then impress it upon the minds of our patients, that while we are willing to make for them a set of teeth on celluloid or rubber, we are not willing to recommend it to be more than it is, viz.: A base used as a substitute for the metals on account of its cheapness, but not on account of its superiority. Some may ask what objection have you to celluloid ; that contains no mercury. Very true, but in my opinion, that is not the objectionable feature; it is the non-conductability of the material. I have only one thing to say in favor of cheap bases, and it is this: That a more accurate fit can be obtained by moulding than by swedging. Gold is beyond all question one of the very best materials for our purposes, taking all things in consideration that the present condition of science has revealed to us. It is not acted upon by any acids that may enter the mouth—is never unpleasant to the taste—will neither discolor nor corrode, and being an excellant conductor, it seldom produces irritation.

You can make it of any degree of thinness consistant with strength, thus interfering in a very slight degree with the articulation. It can be made into full or partial sets of teeth, with or without clasps, and with the use of rubber or celluloid attachments, is readily constructed, making a clean, serviceable, and desirable plate.

The number of dentists scattered over the land who are competent and prepared to do metallic work are not as numerous as you may imagine. 'Tis by practice we become perfect, and there are but few of us who are overrun with this kind of work. Some may say that our colleges afford the student the advantage of becoming adapts in the construction of metallic plates—very true, but after leaving college he is not much troubled with metals, and his knowledge of them only tends to create disgust for the vegetable bases. I consider it one of the important factors in our success as individual practitioners, and the most important one for the good of those to whom we offer our services and to whom we are under obligations for the only success we can achieve, to adhere to metallic work, whereby the natural teeth are more likely to be preserved. And this is our aim and our desire. If not preserved they will then be replaced by one of the very best substitutes that the ingenuity of man has ever devised. And now, gentlemen, let us all make an effort to advance the cause of Mechanical Dentistry, and lift it from its degraded position. If possible, let our motto be no vegetable base except for those whose condition will not admit of better. We should supply a man with what his necessities demand, but let us never seek to make the false impression that a vegetable base is as good as a metallic one.

